# Computerised cognitive remediation to enhance mobility in older adults: a single-blind, single-centre, randomised trial

**DOI:** 10.1016/s2666-7568(21)00173-2

**Published:** 2021-09-02

**Authors:** Joe Verghese, Jeannette R Mahoney, Emmeline Ayers, Anne Ambrose, Cuiling Wang, Roee Holtzer

**Affiliations:** **Department of Neurology** (Prof J Verghese MBBS, J R Mahoney PhD, E Ayers MPH, C Wang PhD, Prof R Holtzer PhD), **Department of Medicine** (Prof J Verghese), **Department of Rehabilitation Medicine** (A Ambrose MD), **and Department of Epidemiology and Population Health** (C Wang), **Albert Einstein College of Medicine, New York, NY, USA; Ferkauf Graduate School of Psychology, Yeshiva University, Bronx, NY, USA** (Prof R Holtzer)

## Abstract

**Background:**

Decline in executive functions and related cognitive processes is associated with mobility decline, and these functions might be amenable to cognitive remediation. This study aimed to examine whether a computerised cognitive remediation programme would improve walking in adults aged 70 years and older.

**Methods:**

This single-blind, randomised trial at one academic centre in the USA evaluated the efficacy of an 8-week computerised programme (also known as brain games) of progressive intensity and complexity to improve walking in older adults at high-risk for mobility disability. Inclusion criteria included being 70 years or older; ambulatory; and at high-risk for mobility disability, defined using a cutscore of nine or less (frail range) on the Short Physical Performance Battery and a walking speed of 100 cm/s or less. Individuals with dementia, acute or terminal medical illnesses, recent or planned surgery affecting mobility, mobility limitations solely due to musculoskeletal limitation or pain that prevented them from completing mobility tests, and those who were nursing home residents were excluded. Participants were block randomised (1:1; block size 12 and no stratification) to the intervention group or the control group (low complexity computer games and health education classes). Primary outcomes were change in walking speed at normal pace and walking while talking conditions assessed from baseline to 8 weeks post-intervention by investigators who were masked to group assignment. Groups were compared using the intention-to-treat principle with linear mixed models adjusted for confounders. This trial was registered with ClinicalTrails.gov, NCT02567227.

**Findings:**

Between March 1, 2016, and March 12, 2020, 383 patients were enrolled and randomly assigned to the intervention or control group. After randomisation, 11 (3%) patients were diagnosed with dementia. 372 (97%; 271 [73%] women) were included in the intention-to-treat analysis. The mean age of participants was 77·0 years [SD 5·6]). 183 (49·2%) participants were Black and 62 (16·7%) were Hispanic. 314 (93%) of the target 338 completers had finished the intervention when the trial was terminated due to the COVID-19 pandemic. Although there were significant within-group improvements in both groups after the 8-week intervention, there was no significant difference in normal walking speed (−1·03 cm/s [SD 1·30]; 95% CI −3·60 to 1·54) and walking while talking conditions (0·59 cm/s [SD 1·61]; 95% CI −2·59 to 3·76) between the intervention and control groups. Similarly, within-group, but no between-group, differences were seen on executive function tests and physical function. There were no severe adverse events related to interventions.

**Interpretation:**

Computerised cognitive remediation improved walking in adults aged 70 years and older at high-risk for mobility disability, but improvements were not significantly greater compared with an active control. Although our findings corroborate the within-group improvements on cognition and mobility reported in previous pilot clinical trials, future studies are required to determine the optimal dose, frequency, intensity, and content of computerised cognitive remediation programmes.

**Funding:**

National Institute on Aging.

## Introduction

The prevalence of mobility disability increases with age.^1^ People with mobility disabilities are less likely to remain in the community, and have high rates of morbidity and mortality.^1–3^ Although a large amount of research supports the role of physical exercise to prevent mobility disability, participation and adherence is low in older adults.^4^ Therefore, it is crucial to explore other approaches to improve mobility in older adults.

Executive functions are a set of higher cognitive processes that modulate behaviour and help individuals manage life tasks and achieve goals. Executive functions and related cognitive processes—such as speed of processing, divided attention, and visuospatial skills—regulate mobility,^5–7^ and impairments in these processes are associated with mobility loss and falls.^5–7^ Medications targeting these cognitive processes have been shown to improve mobility.^8^ Cognitive remediation also improves executive functions and related processes.^9,10^

Computerised cognitive remediation programmes (also known as brain games) are widely available and are marketed to the public to improve cognition, but the evidence base to support prescription of this popular intervention is scarce. In a small pilot study, we reported that a computerised cognitive remediation programme improved speed during a complex walking task in adults aged 70 years and older compared with usual care.^11^ A meta-analysis of cognitive remediation pilot studies concluded that cognitive remediation improved mobility in adults aged 60 years and older who did not have major cognitive, psychiatric, neurologic, or sensory impairments, especially during challenging walking conditions, such as walking while reciting alternate letters of the alphabet.^12^ Despite these promising results, previous cognitive training trials for cognitive or mobility outcomes exhibited limitations including small sample sizes,^11–13^ absence of active controls,^12,14,15^ not accounting for confounders (such as mood or physical activity),^10,12,15–17^ or under-representation of minority ethnic populations.^12,15,18^

Therefore, we aimed to assess the efficacy of a computerised cognitive remediation programme in adults aged 70 years and older at high risk for mobility disability. We hypothesised that cognitive remediation would improve walking performance by improving executive functions related to mobility.

## Methods

### Study design and participants

We did a single-blind, single centre, randomised trial that compared cognitive remediation with an active control. The study design, intervention, and statistical analysis plan have been reported previously,^3,11^ and the design was guided by feedback provided by participants in our pilot study.^3^

Individuals who were 70 years or older; ambulatory; able to speak sufficient English to understand the cognitive assessment battery questionnaire; at high-risk for mobility disability, defined using a score of nine or less (frail range) on the Short Physical Performance Battery (SPPB); and had a walking speed of 100 cm/s or less were eligible for inclusion.^3,11^ Exclusion criteria were having dementia (previous diagnosis or diagnosed based on clinical and neuropsychological test performance at baseline); acute or terminal medical illnesses (eg, cancer [late stage or metastatic disease or receiving active treatment], chronic pulmonary disease on ventilator or continuous oxygen therapy, or active liver disease); recent or planned surgery or hospitalisation for a cause that might affect mobility; mobility limitations solely due to musculoskeletal limitations or pain that might prevent completion of mobility tests; any medical condition or chronic medication use (eg, neuroleptic) that would compromise safety or cognitive function; life expectancy of 12 months or less; severe auditory or visual loss; any active psychoses that would prevent completion of study protocol; having progressive, degenerative neurological disease (eg, Parkinson’s disease or amyotrophic lateral sclerosis); and being resident in a nursing home.^3,11^ Presence of arthritis was not an exclusion criteria if participants could complete the mobility tasks.

Participants were identified from commercially available voter registration lists and electronic clinical databases from the Montefiore health system (New York, NY, USA). The protocol was amended on July 17, 2017, to include additional recruitment sources, such as physician referrals, community events, and advertisements at local clinics and businesses. Screening was done in two phases. A letter explaining the study was sent to all potentially eligible individuals, followed by a telephone interview. Individuals expressing interest were screened via telephone with the Memory Impairment Screen and the AD8 Dementia Screening Interview to exclude individuals with dementia.^3,11^ Potential participants who met eligibility criteria via telephone screening were invited for in-person screening at our academic centre (Albert Einstein College of Medicine, New York, NY, USA). At the second in-person interview, mobility tasks (gait speed and SPPB), medical history questionnaires, and cognitive tests to determine eligibility were done by research assistants. All participants were provided transportation to and from their homes to our centre to avoid inadvertently increasing mobility levels.

The Einstein institutional review board (Albert Einstein College of Medicine) approved the study protocol. All participants gave written informed consent, and the study was done in accordance with the Declaration of Helsinki. The trial was overseen by an independent external data and safety monitoring board.

### Randomisation and masking

The study statistician (CW) generated a block randomisation sequence with a block size of 12 using SAS PROC PLAN (version 9.4) to randomly assign participants (1:1) to the computerised cognitive remediation programme (intervention group) or active control group once participants provided informed consent and completed all baseline assessments.

Randomisation was not stratified. The study statistician did not participate in administering interventions or assessments. The research assistants who enrolled participants were masked to random assignment of each participant until after assignment. The outcome assessor did not participate in administering the interventions and was masked to group assignment. Research assistants did not reveal details of the interventions to participants in the other group. The intervention and control group sessions were done on separate days so that participants in one group were not aware of the specific nature and features of the interventions in the other group.

### Procedures

CogniFit (CogniFit, San Francisco, CA, USA), a commercially available software, provides training across multiple cognitive domains, including executive functions and other related processes—such as speed of processing, divided attention, and visuospatial skills—that are relevant to walking performance.^3,11,18^ Previous studies have suggested that training with CogniFit might improve walking speed.^3,12,18^

For the intervention group, groups of up to eight participants attended three training days per week for 8 weeks. Each session was about 50 min (150 min weekly) and was split into three blocks. The first two blocks of each session included training tasks on multiple cognitive domains and one assessment task that monitored performance and increased task difficulty for training tasks in future sessions. The third block specifically targeted training for executive functions and related processes. Each participant’s training plan was individualised on the basis of the distribution of test scores from their baseline CogniFit cognitive assessment done during session one.^3,11,18^ The quantitative scores for each cognitive domain on the baseline cognitive assessment was used to determine the order of the training activities. Difficulty level was systematically increased across the 8-week training programme based on each individual’s training improvements that were continuously assessed during each training session’s assessment task. CogniFit uses an algorithm to decide which cognitive abilities and tasks should be trained in each session. The algorithm weighs the cognitive ability score from the previous session, which the participants could access, how important the task is for training a specific cognitive ability, how much time has passed without training other cognitive capacities, and other factors. This process ensures that all abilities are trained, but that training intensity is higher for those abilities that require the most training. The algorithm rotated training tasks from a pool of 33 tasks in a way that maximised exposure to tasks that trained the participants’ weakest areas first while systematically introducing tasks that trained areas for which patients obtained a high baseline score to increase overall training performance.

The investigators did not control the order of tasks or difficulty within or across tasks, which was automatically assigned by CogniFit. All participants in the intervention group were trained on all tasks but the order in which participants received the tasks varied. Participants were instructed to complete the tasks as accurately and as quickly as possible. Tasks were designed to be game-like so that they would be enjoyable and would facilitate adherence to the training protocol. Descriptions of the 33 games in the programme are provided in the appendix (pp 1–2).

For the control group, participants also trained for about 150 min every week for 8 weeks. Each session consisted of interactive computer-based health education classes and a low complexity, non-progressive programme designed by CogniFit for this study. The active control computer game included six of the 33 CogniFit games that were used in the intervention group (appendix pp 1–2); the six games did not progress in level of difficulty within or across sessions. The control game programme was designed to resemble the cognitive training programme used by the intervention group. For example, the graphic interface and baseline evaluations were identical in both the control and intervention programmes. CogniFit tasks that specifically targeted executive functions were purposefully excluded from the control group programme. No additional training was done. Each session was divided approximately equally between the health education classes and the CogniFit games. Control sessions were done in similar sized groups and on the same computers as the intervention group, but on different days of the week.

All outcomes were planned to be assessed at baseline, a week after the end of the 8 week intervention, and at 6 and 12 months by a research assistant who did not participate in the interventions and who was masked to group assignment. However, due to the COVID-19 epidemic, some week 9 and 6 month assessments and all 12 month assessments were not possible. At the 8 week post-intervention assessment, to assess effectiveness of masking procedures, participants in both groups were asked to rate if they enjoyed their training and whether they perceived receiving any benefit from the training programme, with both ratings done on a scale of one (worst) to ten (best).

Walking speed at normal pace and walking while talking was done in counterbalanced order on a 20-foot instrumented walkway (GAITRite, CIR systems, Franklin, NJ, USA), and they were only assessed once at each timepoint.^3,11,19^ None of the participants used any assistive devices during the assessments (eg, walking sticks). Walking speed is considered a marker of health and predicts adverse geriatric outcomes.^2,6,20^ Walking speed is accepted as a functional outcome measure for pharmacological trials by the US Food and Drug Administration because of its good validity, reliability, and sensitivity to change.^20^ During walking while talking assessments,^2^ participants walked while reciting alternate letters of the alphabet. The walking while talking test is widely studied as a real-world test of divided attention, and has excellent reliability and validity for predicting falls, frailty, and disability.^21^ Alternate forms of the walking while talking assessment (ie, using different letters) were used to reduce practice effects.^22^

Prespecified baseline covariates included in models were age, sex, years of education, comorbidity score, presence of mild cognitive impairment, pain (assessed with the Medical Outcomes Study pain scale), and cardiac fitness (assessed with the Duke Activity Status Index).^11^ Presence of ten physician-diagnosed illnesses were summed to obtain a comorbidities score (range 0–10): hypertension, myocardial infarction, heart failure, angina, diabetes, depression, chronic lung disease, stroke, Parkinson’s disease, and arthritis.^19^ Mild cognitive impairment and dementia were diagnosed using established criteria by a licensed neuropsychologist (RH), who was masked to group assignment, after reviewing all clinical and neuropsychological data.^3,19,23,24^ Participants who cleared the baseline dementia screening but met criteria for dementia at the second in-person screening assessment were excluded. Tests of general mental status (Blessed Information-Concentration-Memory test),^25^ self-esteem (Rosenberg Self-Esteem Scale), social desirability (Marlowe-Crowne Social Desirability Survey), and social indices (Medical Outcomes Study social support survey and Social Networks Index) were done at baseline.^3,11^ Participants were asked whether they used computers for any purpose and whether they had ever received computerised cognitive training.

We measured potential confounders, including physical activity (assessed with the Community Healthy Activities Model Program for Seniors scale that measures weekly frequency of moderate-intensity exercise-related activities),^26^ frequency of weekly participation in cognitive leisure activities excluding the study sessions (documented with a validated leisure activity questionnaire developed in a sample of community-dwelling older adults in New York, NY, USA),^24^ fear of falls (Activities-specific Balance Confidence and Falls Self-Efficacy scales), and self-reported walking distance in 1 h (were less than a quarter of a mile, half a mile or less, 1 mile or less, 1–2 miles, 2 miles or more).^3,11^ Increased social interactions during the trial might influence cognition and mood, and therefore depressive symptoms (using the Geriatric Depression Scale), anxiety (using the Beck Anxiety Inventory), and quality of life (using the 12-item Short Form Health Survey) with mental and physical components were also assessed at 8 weeks post-intervention.^3^ A subsample of both groups wore Actical accelerometers (Philips, Amsterdam, Netherlands) during the first and last weeks of the trial, and average daily step count was recorded to assess whether amount of walking had changed, which could have affected walking speed.

### Outcomes

The coprimary efficacy outcomes were change from baseline in walking speed at normal pace and walking while talking conditions.

Secondary outcomes included immediate post-intervention change in SPPB^27^ and executive function tests (trail making test B, digit symbol substitution test, letter fluency test) as well as durability of effects (walking speed assessments at 6 and 12 months).^3^ Alternate forms of the executive function tests were used to reduce practice effects. SPPB was designated as a secondary outcome because it includes normal pace walking speed (primary outcome) as one of its components.^3^ Although we proposed to assess neuroplasticity as a secondary outcome using functional near infra-red spectroscopy,^3^ sufficient assessments were not done due to logistical issues such as equipment problems or failures. Some other secondary (gait domains other than speed, stair climbing time, disability scale, and Flanker test) and tertiary (falls) outcomes were also not reported because of additional processing requirements, incomplete or insufficient assessments, or trial suspension restricting follow-up.^3^ A full list of secondary and tertiary outcomes and other study measures are available elsewhere.^3^

Adverse events (such as falls and changes in participants’ health) were recorded by research assistants at study visits and by telephone contact every 2 months following completion of the intervention for up to 12 months. Any serious or potential intervention-related adverse events were reported as they occurred to the data and safety monitoring board. All adverse events were reviewed by the data and safety monitoring board on a biannual basis and by the Einstein institutional review board annually.

### Statistical analysis

Analyses were done with SAS (version 9.4). On the basis of effect sizes from previous trials and from our pilot study,^11,12,17^ we calculated that 169 completers in each group (338 total) would provide 80% power with an α level of 5% to show a difference between groups on change in primary outcomes post-intervention. We inflated the sample size for an estimated 20% dropout rate, resulting in a target sample of 420 participants. In March, 2020, the trial was indefinitely suspended due to the COVID-19 pandemic. The data and safety monitoring board terminated the study on Aug 1, 2020, due to our target almost being met, with 92% of the target sample recruited, and due to infection risk and lifestyle changes during the pandemic.

We used linear mixed effects models adjusted for the prespecified covariates to compare changes in speed during both walking conditions immediately post-intervention and at 6 months after the intervention, according to the intention-to-treat principle. The linear mixed effects model can handle missing data due to dropout missing at random. Sensitivity analyses were done to compare the primary outcomes in the intervention group with the control group, stratified by sex or mild cognitive impairment syndrome status, adjusted for Blessed test scores (general mental status)^25^ as a covariate, or including duration of training as an interaction term in separate models. These additional analyses were adjusted for all prespecified covariates. Similar fully adjusted models were used to examine secondary outcomes, using robust empirical variance estimates (using the sandwich-type estimator of the variance-covariance of the fixed effects parameters) for outcomes with skewed distributions. Difficulty in walking more than a quarter of a mile in 1 h (binary outcome) was examined using generalised linear mixed effects models adjusted for all prespecified covariates using the logit link. The significance level was set at 0·05. The trial is registered on ClinicalTrails.gov, NCT02567227.

### Role of the funding source

The funder had no role in study design, data collection, data analysis, data interpretation, or writing of the report.

## Results

1245 individuals were assessed for eligibility. From March 1, 2016, to trial termination on Aug 1, 2020, 383 (31%) participants were randomly assigned to the intervention (192 [50%] participants) or the control (191 [50%] participants) group. 11 (3%) of participants were diagnosed with dementia after randomisation, so 372 (97%) participants without dementia were included in the intention-to-treat analysis. Of these 372 participants, 314 (84%) participants completed the immediate post-intervention assessment, and 240 (65%) completed the 6-month assessment (figure). Following random assignment, two (1%) of 186 participants in the intervention group received physical therapy, four (2%) of 186 participants in the control group received the intervention programme, and 13 (3%) of 372 participants altered their training schedule due to personal reasons to complete the 8-week programme in 5–7 weeks (12 [6%] participants in the intervention group and one [1%] participant in the control group). 11 (6%) participants in each group did not complete their programme because of trial suspension due to the COVID-19 pandemic. Because of the COVID-19 pandemic, we could not examine durability of effects at 1 year as only 175 participants had completed the 12-month assessment when the trial was suspended. All participants were analysed in their originally assigned groups using the intention-to-treat principle.

For the 372 participants included in the intention-to-treat analysis, mean age of participants was 77·0 (SD 5·6) years. 271 (73%) were women, 183 (49%) were Black, and 62 (17%) were Hispanic (table 1). This sample had a high prevalence of mild cognitive impairment, slow walking speeds, and low levels of cognitive and physical activity participation (table 1). 111 (60%) of 186 participants in the intervention group and 115 (62%) of 186 participants in the control group reported using a computer (any frequency) over the past year. No participants reported current or past cognitive training.

The median duration of training in the intervention group was 997·4 min (IQR 996·0–1235·5), and the control group received a median of 1050·8 min (1054·5–1210·2) of health education and cognitive training. Median attendance in the intervention group was 83·3% (IQR 62·5–91·6) and 100% (87·5–100·0) in the control group. On a scale of one (worst) to ten (best), participants in both groups had a median response of ten (IQR nine to ten) when asked how much they enjoyed the sessions. Both the intervention (nine [eight to ten]) and control groups (ten [eight to ten]) reported that they benefited from the programme.

Compared with baseline at the post-intervention assessment at 8 weeks, normal pace walking speed increased in the intervention (1·73 cm/s [SD 0·94]; p=0·065) and the control group (2·76 cm/s [0·91]; p=0·003), but there was no significant difference between control and intervention groups in the fully adjusted models (−1·03 cm/s [95% CI −3·60 to 1·54]; p=0·43). Walking while talking speed also increased in the intervention (6·02 cm/s [SD 1·16]; p=0·0001) and the control group (5·43 cm/s [1·13]; p=0·0001), but there was no significant between-group difference in the fully adjusted models (0·59 cm/s [95% CI −2·59 to 3·76]; p=0·72).

There were no significant differences between the intervention and the control groups for the primary outcomes in the fully adjusted models when stratified by sex or mild cognitive impairment status, when adjusted for Blessed test scores,^26^ or when duration of training was included as an interaction term (data not shown).

After the 8 week intervention, both groups showed within-group improvements in SPPB. Within-group improvements were also observed in all three executive function tests in the intervention group and in two executive function tests in the controls, but there were no significant differences between the intervention and control groups for any of the secondary endpoints (table 2).

For the 240 participants (112 [47%] from the intervention group and 128 [53%] from the control group) who completed the 6-month assessment, there were no significant differences between the intervention and the control groups for normal walking speed (1·13 cm/s, [95% CI −1·65 to 3·91]; p=0·48) or walking while talking conditions (1·40 cm/s [95% CI −2·12 to 4·91]; p=0·42) in the fully adjusted models.

At the 8-week post-intervention assessment, there were no significant between-group differences in physical or cognitive leisure activity levels, self-reported mobility difficulty, fear of falls, depressive symptoms, anxiety symptoms, or quality of life (table 3). 299 (80%) of 372 participants had accelerometery data; there were no significant differences between the intervention and the active control group in daily step count post-intervention (−553·5 [SD 522·9]; 95% CI −1583·4 to 476·4; p=0·83).

No serious adverse events related to the study were adjudicated by the data and safety monitoring board. Four (1%) participants died, but the deaths were unrelated to the study (three in the intervention group and one in the control group). In the intervention group, 57 (31%) of 186 participants reported adverse events outside of training (up to 12 months): four (2%) had high blood pressure, 15 (8%) had leg pain, five (3%) had arthritis, and 33 (18%) had falls. In the controls, 50 (27%) of 186 participants reported adverse events outside of sessions: two (1%) had high blood pressure, seven (4%) had leg pain, seven (4%) had arthritis, and 34 (18%) had falls.

## Discussion

In this single-blind randomised controlled trial, an 8-week computerised cognitive remediation programme of progressive intensity and complexity did not improve walking, physical performance or executive functions in adults aged 70 years and older at high risk for mobility disability compared with an active control programme consisting of computerised health education classes and low-intensity and non-progressive computer games. But our findings corroborate findings of previous clinical trials showing within-group improvements in walking and cognition following cognitive training in older adults of similar age to the current sample.^3,9,14,15,17^ To our knowledge, this is the first large-scale randomised clinical trial with an active control to examine far transfer effects of cognitive remediation on mobility outcomes.

Our findings that computerised cognitive remediation was not more efficacious than the active control in improving mobility and cognition was unexpected because our pilot study and meta-analysis of cognitive interventions had shown mobility benefits of cognitive remediation.^11,12^ Several explanations for this discrepancy in findings should be considered. First, practice effects, defined as improvement in test performance due to repeated evaluation with the same test materials,^21^ might lead to improvement in both the intervention and control groups. Large practice effects were observed in the no-contact control group of the Advanced Cognitive Training for Independent and Vital Elderly (ACTIVE) trial,^15^ with 31% of participants showing a reliable improvement in reasoning and 32% an improvement in processing speed at 12 months. Practice effects might account for within-group improvements on our primary walking outcomes. But practice effects do not explain why the estimates of within-group change were higher for two out of three executive function tests in the intervention group compared with the control group, despite the same number of test administrations in both groups, which were also well balanced on baseline characteristics. Moreover, we used alternate forms of the walking while talking assessment to reduce practice effects on this assessment and on executive function tests.^22^ Second, a true training effect in both groups needs to be considered. Because cognitive and physical activity participation is often low in older adults,^24,25^ even low-intensity cognitive training in a group setting with socialisation might be beneficial. Hence, the training programme used in the control group might have been sufficient to induce near transfer effects in cognition, though effects were stronger in the intervention group that received the higher-intensity training. The similar mobility and cognitive effects within the intervention and control groups might reflect the similarity in the computerised training programmes. Cognitive gains have been reported in adults without dementia with increasing frequency and duration of training and complexity of cognitive training programmes.^15^ The ACTIVE study reported within-group improvements in executive function even 2 years after 10 h of cognitive training.^9^ In a pilot study of 78 healthy adults aged 60 years and older, training on executive function tasks did not increase walking speed, but it did improve performance on the Stroop test, which was included in the training programme.^28^ Finally, cognitive remediation might need to be combined with physical exercises to produce far transfer effects to mobility. Previous studies suggest that combined physical and cognitive training improves cognition but effects on mobility and walking are not conclusive.^16,29^ Shatil^18^ reported that 62 healthy adults aged 65–93 years who engaged in the CogniFit programme (alone or in combination with aerobic exercises) over 4 months showed improvement in tests of executive function and other cognitive domains compared with 60 adults who did only aerobics or supervised reading over the same period. Of note, mobility and functional outcomes were not examined in this previous study.^18^ Future studies should consider the types of physical and cognitive training programmes to be combined for synergistic effects on walking. A pilot clinical trial in 90 healthy adults aged 60 years and older showed that a 3-month computer lesson programme improved walking speed when combined with aerobic and resistance exercises, but not when combined with stretching exercises.^13^ There is a need for more well designed clinical trials to address the efficacy of the combined cognitive and physical approach on mobility.

Our trial had certain strengths. We focused on secondary prevention and enrolled a population at increased risk for mobility loss.^1,2,6^ There was very high adherence to the training, and participants in both groups reported high levels of enjoyment and benefits from the programmes. We used validated and clinically relevant outcomes. Ethnic and racial differences in walking speeds have been previously reported;^1^ most participants in our study were from racial and ethnic minority backgrounds, whereas previous trials recruited mostly or only White participants.^12,15,17,30^ We applied the intention-to-treat principle, which includes all randomly assigned participants, rather than just those who completed the trial. Previous cognitive training trials, in isolation or combined with physical exercise, reported improvements in cognition.^9,15,17,30^ But the comparator groups in most clinical trials were usual or no-treatment groups^,15,17,30^ or passive control conditions, such as watching educational videos.^17^ Unlike these trials, we used an active control that was not only matched to the intervention group for level of computer exposure and social interactions but also included low intensity and non-progressive computerised cognitive training for approximately half of the control session. But the similar within-group improvements for both primary and secondary outcomes in both study groups raise the possibility that the additional executive function training in the intervention group might not be necessary or was not sufficiently robust to induce stronger far transfer effects to mobility, compared with the control group. We addressed several important confounders not considered in previous clinical trials, which might explain improvements in the intervention condition compared with passive or no control conditions in these trials.^12,15,29,30^ Our results showed no group differences in self-esteem, social desirability, or social indices at baseline. There were no post-intervention changes in fear of falls, mood, cognitive or physical leisure activity levels, or quality of life metrics within or between groups. Accelerometry data showed no between-group differences in step counts over the study period.

However, our study had limitations. The trial was stopped early due to the COVID-19 pandemic, but we were close to reaching our target of 338 completers: 314 (92·8%) participants completed the trial before termination of the trial. Hence, inadequate power is unlikely to account for negative results. An even larger sample might have shown small between-group differences, which might have been statistically but not clinically significant. Delayed intervention effects might have been missed due to early trial termination. Most participants (73%) were women. Women appear to show more cognitive benefits in exercise trials,^31^ but a sex difference in response to cognitive training was not clearly established by previous studies. The intervention in our trial was longer than that in many previous trials,^15,17,30^ but even longer or more intense training might be needed to show between-group differences that would support far transfer effects of cognitive remediation to mobility. Although effects of other cognitive training programmes on neuroplasticity have been reported using neuroimaging and neurophysiological techniques,^32^ to our knowledge, no studies have specifically examined neuroplasticity effects of the CogniFit programme.

In conclusion, a computerised cognitive remediation, or brain games, programme for secondary prevention of mobility loss in adults older than 70 years at high risk for mobility disability did not have far transfer effects in improving walking in simple and complex conditions compared with an active control programme. However, the within-group improvements in walking and cognitive outcomes in both the intervention and control groups raise the possibility of either practice or training effects that warrants examination.

## Supplementary Material

1

## Figures and Tables

**Figure:**
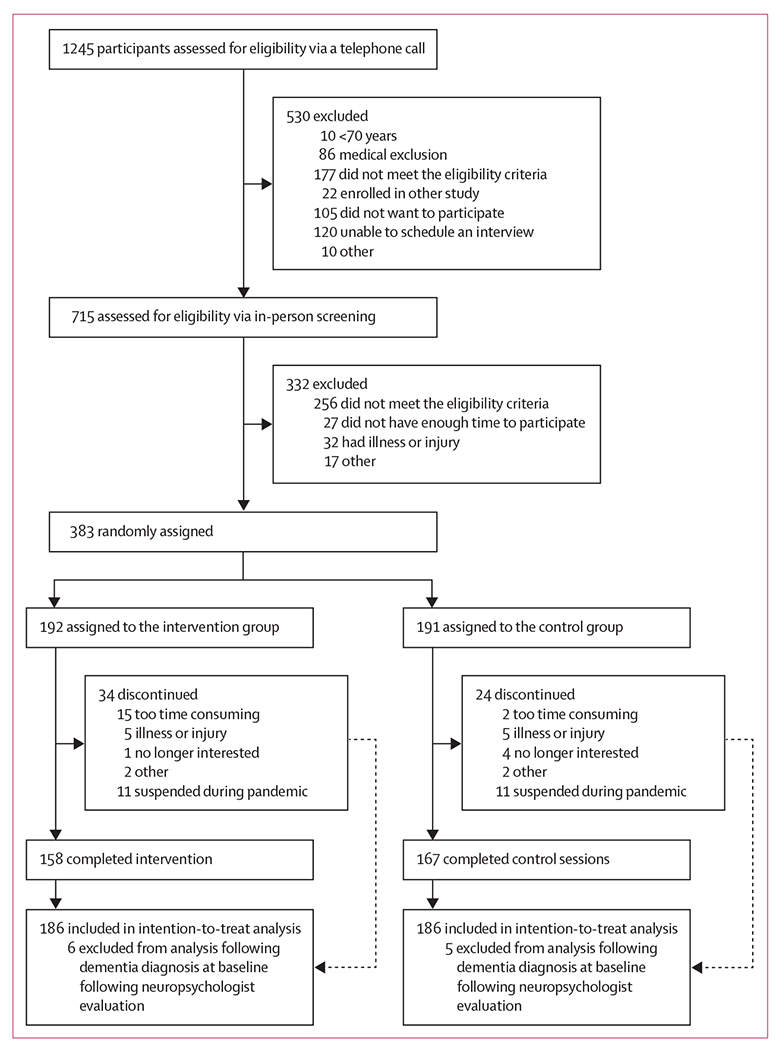
Trial profile

**Table 1:** Baseline characteristics of intervention and control groups

	Intervention (n=186)	Active control (n=186)
Mean age, years	76·9 (5·7)	77·1 (5·6)

Women	135 (73%)	136 (73%)

Men	51 (27%)	50 (27%)

Race or ethnicity		
White	57 (31%)	27 (25%)
Black	93 (50%)	80 (48%)
Hispanic	28 (15%)	34 (18%)
Asian	1 (1%)	5 (3%)
Other and unknown	7 (4%)	10 (5%)

Mean years of education	14·08 (3·18)	13·90 (2·93)

Mean comorbidity score (0–10)	1·96 (1·15)	2·18 (1·17)

Mean walking speed at normal pace, cm/s	78·13 (18·29)	77·13 (18·86)

Mean walking while talking speed, cm/s	53·44 (18·79)	53·58 (18·63)

Mean Short Physical Performance Battery score (0–12)*	6·43 (1·76)	6·47 (1·86)

Mild cognitive impairment	65 (35%)	58 (31%)

Mean Duke Activity Status Index (0–58·2)*†	32·21 (13·18)	33·44 (14·00)

Mean Medical Outcomes Study pain scale (0–6)‡	2·88 (1·45)	2·95 (1·49)

Mean Blessed test score (0–32)†	3·84 (2·36)	3·69 (2·29)

Mean digit symbol substitution test (0–133)*	42·08 (12·66)	42·31 (12·63)

Mean letter fluency test (Normative range for age and education 25–41)*	34·23 (12·12)	34·05 (10·05)

Mean trail making test B (0–300 s)†	172·40 (73·35)	179·83 (77·28)

Cognitive leisure activities, days per week	4·70 (2·81)	4·81 (2·63)

Mean physical leisure activities (CHAMPS), days per month	7·15 (9·33)	7·44 (9·82)

Computer used in past year	111 (60%)	115 (62%)

Mean Marlowe-Crowne Social Desirability Survey score (range 0–33)	24·14 (4·50)	23·95 (4·94)

Mean Rosenberg Self-Esteem Scale (range 0–40)*	34·68 (4·51)	35·43 (4·56)

Mean number of high contact relationships (social support)§	4·83 (1·58)	4·66 (1·75)

Mean social network size (total number of contacts)	17·89 (13·27)	17·09 (11·77)

Data are n (%) or mean (SD). CHAMPS=Community Healthy Activities Model Program for Seniors.

* Higher score represents a better performance.

† Lower score represents a worse performance.

‡ n=184 for the intervention group and n=185 for the control group.

§ Number of people with whom the respondent has contact at least every 2 weeks.

**Table 2:** Summary of post-intervention effect on primary and secondary outcomes

	Intervention group estimate* (n=186)	Control group estimate* (n=186)	Between-group estimate†	Between-group estimate*
**Primary**				

Normal walking (cm/s)	1·73 (−0·11 to 3·57)	2·76 (0·97 to 4·55)	−0·97 (−3·55 to 1·61), 0·46	−1·03 (−3·60 to 1·54), 0·43

Walking while talking (cm/s)	6·02 (3·74 to 8·29)	5·43 (3·22 to 7·65)	0·63 (−2·55 to 3·82), 0·70	0·59 (−2·59 to 3·76), 0·72

**Secondary**				

Short Physical Performance Battery (range 0–12)	0·70 (0·45 to 0·95)	0·85 (0·61 to 1·08)	−0·15 (−0·49 to 0·20), 0·40	−0·15 (−0·49 to 0·20), 0·40
Log trail making test B (range 0–300)‡	−0·10 (−0·15 to 0·05)	−0·10 (−0·15 to−0·05)	−0·00 (−0·07 to 0·06), 0·94	−0·00 (−0·07 to 0·06), 0·93
Letter fluency test, (normative range 25–41)	1·20 (0·10 to 2·30)	0·53 (−0·54 to 1·60)	0·63 (−0·90 to 2·17), 0·42	0·67 (−0·86 to 2·20), 0·39
Digit symbol substitution test (range 0–133)	2·22 (1·28 to 3·16)	1·21 (0·31 to 2·12)	0·97 (−0·33 to 2·28), 0·14	1·01 (−0·30 to 2·31), 0·13

* Estimates with 95% CI adjusted for age, sex, education (school years), comorbidities score (0–10), pain, Duke score (cardiac fitness), and cognitive status (mild cognitive impairment or cognitively normal).

† Unadjusted estimates.

‡ Trail B scores log transformed as distribution was skewed.

**Table 3:** Post-intervention effects on other variables

	Intervention		Active control		Between-group estimate*
	Baseline (n=186)	8 weeks (n=152)	Baseline (n=186)	8 weeks (n=162)	
Mean CHAMPS scale score† (range 0–133)	2·07 (3·31)	1·65 (2·97)	2·26 (3·65)	1·91 (3·17)	−0·09 (−0·64 to 0·46), 0·76

Cognative leisure activities, days/week	4·70 (2·81)	5·06 (2·63)	4·81 (2·63)	5·36 (2·50)	−0·35 (−0·88 to 0·19), 0·20

Mean Falls Self-Efficacy (range 10–100)	15·87 (9·69)	16·2 (9·17)	15·92 (11·09)	16·28 (10·72)	0·39 (−1·38 to 2·16), 0·66

Mean Activity Balance Confidence Scale score (range 0–100)	73·86 (20·84)	75·86 (19·61)	76·72 (21·32)	76·49 (22·67)	1·28 (−1·68 to 4·24), 0·39

Mean Geriatric Depression Scale score (range 0–30)	5·04 (3·99)	5·43 (5·17)	5·11 (4·29)	5·25 (4·83)	0·26 (−0·40 to 0·93), 0·44

Mean Beck Anxiety Inventory score (range 0–63)	4·11 (6·51)	3·92 (7·22)	3·00 (5·19)	2·89 (5·69)	−0·26 (−1·51 to 1·00), 0·84

Walk less than a quarter of a mile in 1 h	25 (13%)	21 (14%)	35 (19%)	32 (20%)	−0·01 (−0·99 to 0·98), 0·98

Mean SF-12 mental health score	55·45 (7·91)	54·65 (8·90)	56·81 (7·13)	55·63 (8·19)	0·41 (−1·04 to 1·85), 0·58

Mean SF-12 physical health score	39·09 (5·95)	39·47 (5·60)	38·84 (6·20)	39·28 (5·84)	−0·21 (−1·46 to 1·04), 0·74

Data are mean (SD), n (%), or adjusted estimates (95% CI), p value. CHAMPS=Community Healthy Activities Model Program for Seniors.

* Estimates with 95% CI and p values are from linear mixed effects models adjusted for age, sex, education (school years), comorbidities score (0–10), pain, Duke score (cardiac fitness), and cognitive status (mild cognitive impairment or cognitively normal).

† CHAMPS scale measures weekly frequency of moderate intensity exercise related activities.

## Research in context

### Evidence before this study

We searched PubMed for articles published from the inception of the database to Jan 1, 2021, using the terms “cognitive remediation” OR “cognitive training” AND “mobility” OR “walking” OR “gait” OR “cognition” OR “clinical trials”. We also screened papers by reviewing abstracts to identify full-text reports that were relevant to our aims. More than 100 clinical trials and observational studies were identified. Evidence from observational and epidemiological studies indicate that executive functions and related cognitive processes are linked to the maintenance of mobility and to the risk of developing mobility disability. Preliminary studies suggest that cognitive remediation could improve walking performance by enhancing executive functions.

### Added value of this study

Previous clinical trials examining the role of cognitive training to improve cognition and mobility were limited by small sample sizes, absence of active controls, and they did not account for confounders (such as mood or physical activity), or under-represented minority populations. To the best of our knowledge, this is the first large-scale randomised clinical trial with an active control (health lessons and low complexity computer games) to examine far transfer effects of an 8-week cognitive remediation programme of progressive intensity and complexity to mobility outcomes. Both the intervention and active control groups showed within-group improvements in walking, physical function, and cognitive outcomes, confirming results of previous studies. However, the cognitive remediation intervention programme did not significantly improve cognitive and walking outcomes compared with the control programme.

### Implications of all the available evidence

Our findings raise questions regarding optimising cognitive remediation approaches in improving cognition and mobility in older adults that should be addressed in future studies. For instance, what are the optimal type, components, and dose of cognitive remediation required to improve outcomes, and should cognitive training be combined with physical exercise?
