# Supplementary material for: Computerised cognitive remediation to enhance mobility in older adults: a single-blind, single-centre, randomised trial
Source: Lancet Healthy Longev. Author manuscript; Available in PMC 2021 Sep 13. (PMC8437150; doi:10.1016/s2666-7568(21)00173-2)
Supplement: 1 [file NIHMS1738034-supplement-1.pdf]

THE LANCET
Healthy Longevity

Supplementary appendix

This appendix formed part of the original submission and has been peer reviewed. We post it as supplied by the authors.

Supplement to: Verghese J, Mahoney JR, Ayers E, Ambrose A, Wang C, Holtzer R. Computerised cognitive remediation to enhance mobility in older adults: a single-blind, single-centre, randomised trial. *Lancet Healthy Longev* 2021; **2:** e571–579.

Appendix 1. Cognifit Games

Game name	Instructions
**Bee Balloon**	Pop all the balloons on the screen. Move through the area without touching the red zones and bombs! You'll only have a certain number of lives before you're brought down a level, so be careful not to waste them!
**Butterfly Hunter**	You will hunt butterflies in the sky. Catch the butterflies as fast as you can and avoid everything else. You can dodge anything when surfing a tornado.
**Candy Factory***	Remember the candy in front of each pumpkin. Memorize both the type of candy and its flavor. When the candy disappears, fill each pumpkin with the same candy as previously shown by dragging the candy from the factory down to the pumpkin.
**Crossroads**	Prevent the rolling balls from colliding. When you see two balls approaching the same intersection, click on that intersection as fast as you can to prevent the balls from colliding. Try not to place unnecessary blocks on the grid.
**Cube Foundry**	You are assembling blocks of various shapes to create continuous layers in a 3D environment. To do so, you can rotate the blocks around all three axes as well as move the blocks horizontally and vertically.
**Dragster Racing***	The goal is to win the race. You must press the space bar to start when the light is green and press again before the arrival line to avoid crashing the vehicle. The vehicle accelerates and brakes automatically.
**Fresh Squeeze**	Bring the Fresh squeeze juice to our adorable and thirsty monster's mouth. To do so, draw a straw path from the Fresh squeeze machine to our friend's mouth. Plan your moves ahead because if you get stuck on the way, the Fresh squeeze will spill! Don't make your paths too short: you'll need to use a minimum number of straws to go to the next level.
**Fuel a Car**	You are managing a gas station. Icons show you what cars need. Click on the corresponding-colored equipment to select the service and then click back on the car. Once the service is completed, click on the gate to let the car through. Click as fast as you can to avoid chaos.
**Gem Breaker***	Your goal is to break brick lines using a paddle to bounce ball back at the blocks without letting the ball touch the bottom of the screen. You control the paddle using your mouse. Press spacebar to launch ball or to fire a missile. Some special blocks contain bonuses such as explosives, extra balls, side quests, etc. or powerups that add extra features to your paddle such as bigger paddle, sticky paddle, missiles, megaballs and many more!
**Gem Breaker 3D**	Your goal is to break brick lines using a paddle to bounce ball back at the blocks without letting the ball touch the bottom of the screen. You control the paddle using your mouse. Press spacebar to launch ball or to fire a missile. Some special blocks contain bonuses such as explosives, extra balls, side quests, etc. or powerups that add extra features to your paddle such as bigger paddle, sticky paddle, missiles, megaballs and many more!
**Jigsaw 9**	Rearrange a jumbled picture by moving its pieces one at a time into empty spaces. You can move a piece by clicking on it. It will then slide into the empty space adjacent to it. Plan your moves ahead so you can complete the picture within the recommended number of steps above.
**Lane Changer**	The goal is to pass all the cars along the highway. You can move through the lanes by pressing on the up and down arrows and you can move faster or slower by pressing on the right and left arrows. The more cars you pass, the more points you will obtain. Be careful not to crash with other vehicles!
**Mahjong**	The aim of the game is to match the tiles with the same image in order to get rid of them. However, the tiles that can be eliminated are only those with free sides around them.
**Math Twins**	Your goal is to select two numbers whose added value equals the number shown in the upper right corner of your screen. You can only pass-through empty cases in a straight path, with only two direction changes available. Tip: you can combine colors and forms to get extra time combos. If you get stuck, you can refresh the grid by pressing the reset button.
**Minus Malus**	Your goal is to shoot crates falling from the sky while solving small calculations. Every crate has a corresponding number. Destroy crates using numbered bullets. The sum of bullets fired must exactly equal the number displayed on the crate. Aim crates using your mouse and fire the right bullet using the keys S, D or F of your keyboard as explained on the upper right corner of your screen. Be careful as you have a limited number of bullets.
**Mouse Challenge**	Click as fast as you can on items scattered on the screen in a specific order. The items and orders vary from one level to another. Sometimes, the cursor behaves strangely and moves in unexpected directions. You are notified of the changes before they occur.

**Neuron Madness**	The goal is to collect all the neurons in the color order shown in the display. To do so you can move your ship along the stage using the arrow keys. Be careful and avoid bombs, obstacles, and the Enemy, as he will eat all the neurons in the stage. There are lots of different elements to play with including portals, conveyor belts, moving neurons and many more.
**Numbers line***	Your goal is to clear the conveyor belt before the tokens fall off in the hole. Aim the token with your mouse and click to shoot the next token. You can see it on the cannon. Tip: you can combine multiple tokens in a row, same color and same form gives you extra time.
**Perfect Tension**	You are playing Tangram, a Chinese puzzle consisting of seven flat shapes, called tans, which are put together to form shapes. Be careful, this Tangram is unusual because gravity will work against you when you move the tans. You must find the perfect balance to solve the puzzle!
**Piece Making**	There will be totem stones presented. Try to remember the symbols on the totem stones. Then, click on the card that includes every totem symbol that you saw before.
**Reaction Field**	Get rid of the moles that appear in your garden by clicking on them. Watch the target color sign and only click on the moles with the same color as fast as you can. Moles with helmets will have to be hit twice instead of once to get rid of them. Do not click on the moles with a dynamite stick strapped on their head as this will result in a penalty.
**Shore Dangers**	The goal is to get objects on the correct side of the shore as fast as possible. Use the mouse to move the gap in the tree-trunk to allow the objects to pass from one side to the other. Rubber rings, balls and leaves belong to the beach. Fish and shellfish belong to the sea.
**Slice and Drop**	The objective of this game is to lead birds to the floor and keep them away from cats. To help birds, you can interact with the surrounding such as ropes and screws or slice wooden shelves by drawing a line with your mouse. However, you cannot slice fixed blocks. Carefully plan your moves as things might be more complicated than they appear at first glance.
**Star Architect**	You are building 3D shapes using the cubes that are sent to you. To do so, you see three walls made of different colored squares. You have to place cubes at the intersection of same-colored squares from each wall. The cube's color must be the same as the squares' color. You can move cubes horizontally using arrows of your keyboard. To confirm the final position of a cube, simply press spacebar.
**Sudoku**	The goal is to fill in all the cells of a grid with digits as fast as possible. Every row, column and sub-grids must contain one of each digit. The number of digits depends on the level.
**Tennis Bomb**	Your goal is to hit tennis balls and avoid bombs. Be careful, only hit balls that match the picture shown on the TV screen. Press the space bar when you see on the TV screen the color that was presented at the beginning.
**Tennis Bowling**	Your goal is to bring barrels down using as few tennis balls as you can. Be careful: do not aim at barrels with flames. Press the space bar to launch the ball at the speed shown on the right indicator.
**Tennis Target**	Your goal is to hit the ball at a target. Be careful, do not aim at boxes which have a flame on them. Use the key arrows of your keyboard to adjust the direction of the ball and press space bar to launch the ball.
**Traffic Manager**	The goal is to control the traffic at different intersections by switching traffic lights from red to green by clicking on them. You need to avoid collisions and traffic jams. To complete the level, you need to pass the quantity of cars shown at the lower right corner of your screen in a limited amount of time!
**Twist It***	The objective of this game is to align three or more pieces of the same kind, horizontally or vertically, by swapping one with the other. You need to align at least three pieces. You can get special pieces by merging four or five pieces. These pieces will let you break more blocks. Finally, the goal is to reach the target pieces shown in the screen before time ends up.
**Water Lilies**	Remember the sequence in which flowers will be highlighted. During your turn, click on the flowers according to the order previously presented. Click "Next" when you are ready to start the game.
**Word Quest***	Look for hidden words in a puzzle. Look at the pictures displayed. Try to match the pictures name with their words by selecting the corresponding letters in the square of random letters as fast as you can. Press the "Hint" button to find the word more easily but avoid using the hint and clicking unnecessary letters.
**Words Birds**	Rearrange a group of birds carrying letters to form a word. The word you must find is represented by a picture at the bottom left corner of the screen. Slide the columns and rows of birds to form the word in the highlighted central row. The faster you find the word, the higher the score that you will receive.

*Only the lowest level of difficulty on these games without progression included in the active control program.
